# Diagnostic accuracy of cerebral [^18^F]FDG PET in atypical parkinsonism

**DOI:** 10.1186/s13550-023-01025-x

**Published:** 2023-08-12

**Authors:** Naba Jawad Houssein, Alexander Cuculiza Henriksen, Anne-Mette Hejl, Lisbeth Marner

**Affiliations:** 1 Department of Clinical Physiology and Nuclear Medicine, Copenhagen University Hospital Bispebjerg, Bispebjerg Bakke 23, Copenhagen, Denmark; 2 Faculty of Health and Medical Sciences, University of Copenhagen, Copenhagen, Denmark; 3 Department of Neurology, Copenhagen University Hospital Bispebjerg, Copenhagen, Denmark

**Keywords:** Positron emission tomography, Neurodegenerative disorders, Progressive supranuclear palsy, Lewy body dementia, Multiple system atrophy, Corticobasal degeneration, Sensitivity, Specificity

## Abstract

**Background:**

Atypical parkinsonism (AP) often presents with Parkinson’s symptoms but has a much worse long-term prognosis. The diagnosis is presently based on clinical criteria, but a cerebral positron emission tomography (PET) scan with [^18^F]fluoro-2-deoxy-2-d-glucose ([^18^F]FDG) may assist in the diagnosis of AP such as multiple system atrophy (MSA), progressive supranuclear palsy (PSP), corticobasal degeneration (CBD), and Lewy body dementia (DLB). Only few studies have evaluated the sensitivity and specificity of [^18^F]FDG PET for separating the diseases in a mixed patient population, which we aim to assess in a retrospective material.

**Results:**

We identified 156 patients referred for a cerebral [^18^F]FDG PET for suspicion of AP during 2017–2019. The [^18^F]FDG PET was analysed by a nuclear medicine specialist blinded to clinical information but with access to dopamine transporter imaging. The reference standard was the follow-up clinical diagnosis (follow-up: 6–72 months). The overall accuracy for correct classification was 74%. Classification sensitivity (95% confidence interval, CI) and specificity (95% CI) for MSA (n = 20) were 1.00 (0.83–1.00) and 0.91 (0.85–0.95), for DLB/Parkinson with dementia (PDD) (n = 26) were 0.81 (0.61–0.93) and 0.97 (0.92–0.99) and for CBD/PSP (n = 68) were 0.62 (0.49–0.73) and 0.97 (0.90–0.99).

**Conclusions:**

Our results support the additional use of [^18^F]FDG PET for the clinical diagnosis of AP with moderate to high sensitivity and specificity. Use of [^18^F]FDG PET may be beneficial for prognosis and supportive treatment of the patients and useful for future clinical treatment trials.

## Background

Parkinson’s disease affects approximately 1% of individuals over the age of 65. Among them 75% have idiopathic Parkinson’s disease (IPD), while the remaining 25% have atypical syndromes, including symptomatic parkinsonism [1]. Some patients initially exhibit symptoms of Parkinson’s disease but quickly progress to more severe and potentially life-threatening symptoms. These are classified under atypical parkinsonism (AP), which encompasses: Dementia with Lewy Bodies (DLB); Progressive Supranuclear Palsy (PSP), Multiple System Atrophy (MSA) and Corticobasal Degeneration (CBD). The incidence rate of AP is 2.5–5.9 per 100.000 person-years and it increases with age. Patients with AP have a mean survival of 1.8–9.5 years from diagnosis [2–4].

Precise AP diagnosis is crucial for accurate prognosis and effective therapy, but the disease is often difficult to diagnose in early stages based on clinical criteria alone [5]. Some studies have employed cerebral [^18^F]FDG PET for improved differential diagnosis of Parkinson’s disease, as it can identify regional metabolism patterns related to each AP subtype [6–8]. However, the sensitivity and specificity of cerebral [^18^F]FDG PET in diagnosing AP remain under-examined, and evidence supporting its routine use is limited [8, 9].

Although some studies report high sensitivity and specificity (> 85%) in diagnosing AP subtypes, these studies primarily focus on selected populations with diagnoses of either AP or IPD [10, 11]. A prospective study on a mixed population found high sensitivity and specificity in distinguishing AP from DLB/PDD [12]. Nevertheless, no studies following the Standards for Reporting Diagnostic accuracy studies (STARD) guidelines have investigated the sensitivity and specificity of cerebral [^18^F]FDG PET in diagnosing AP in a mixed population. This is especially important when considering a diverse patient population reflective of daily clinical routine.

This study therefore aims to assess the diagnostic accuracy of additional cerebral [^18^F]FDG PET in diagnosing AP and its subtypes, including MSA, DLB/PDD and 4-repeat tauopathies (4R-tauopathies), which encompass CBD and PSP, within a mixed population.

## Material and methods

### Ethical approvals

This study received approval from the Danish Patient Safety Authority (31-1521-255) and the Danish Data Protection Agency (P-2020-530).

### Study population

**Fig. 1:**
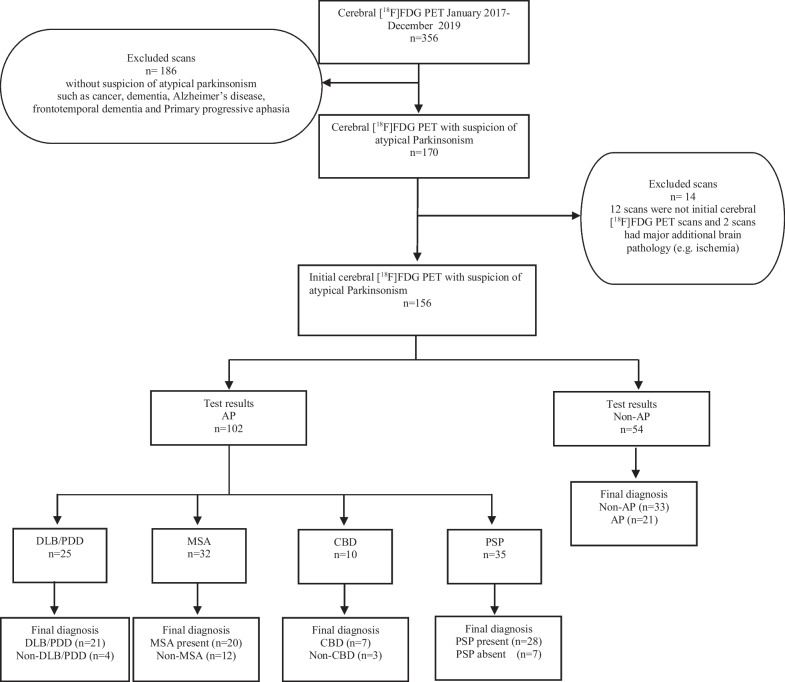
STARD flow diagram. Flow diagram of recruitment and diagnostic classification of study subjects from January 2017 to December 2019 according to the Standards for Reporting of Diagnostic Accuracy (STARD) studies. AP, atypical parkinsonism; Non-AP, atypical parkinsonism not present; DLB/PDD: Dementia with Lewy bodies/Parkinson’s disease with dementia; MSA, multiple system atrophy; CBD, corticobasal degeneration; PSP, progressive supranuclear palsy

All cerebral [^18^F]FDG PET scans patient conducted from January 1st, 2017 to December 31st, 2019 at the Department of Clinical Physiology and Nuclear Medicine after referral from the Department of Neurology at Copenhagen University Hospital Bispebjerg were reviewed. The department is a tertiary movement disorder clinic evaluating approximately 2500 patients annually with diverse movement disorders. This study included patients with suspected Atypical Parkinsonism (AP) and excluded individuals showing initial symptoms or diagnoses of other diseases such as cancer, dementia, or Alzheimer's disease. In cases where multiple cerebral [^18^F]FDG PET scans were available, the first referral indicating the possibility of AP was selected for analysis. Figure 1 provides the flowchart of inclusion.

A specialized physician with expertise in movement disorders clinically evaluated all participants. The Montreal Cognitive Assessment (MoCA) tool was used to measure global cognitive functions. Patients with MoCA scores above 22 underwent additional neuropsychological examinations.

Most patients underwent a Magnetic Resonance Imaging (MRI) scan (87%) and a DAT scan (84%). As part of the initial clinical assessment, 63% underwent a MoCA test and 70% of the patients underwent spinal fluid examinations, including routine tests as well as specific assessments for Alzheimer's disease and Neurofilament Light Chain levels.

**Fig. 2:**
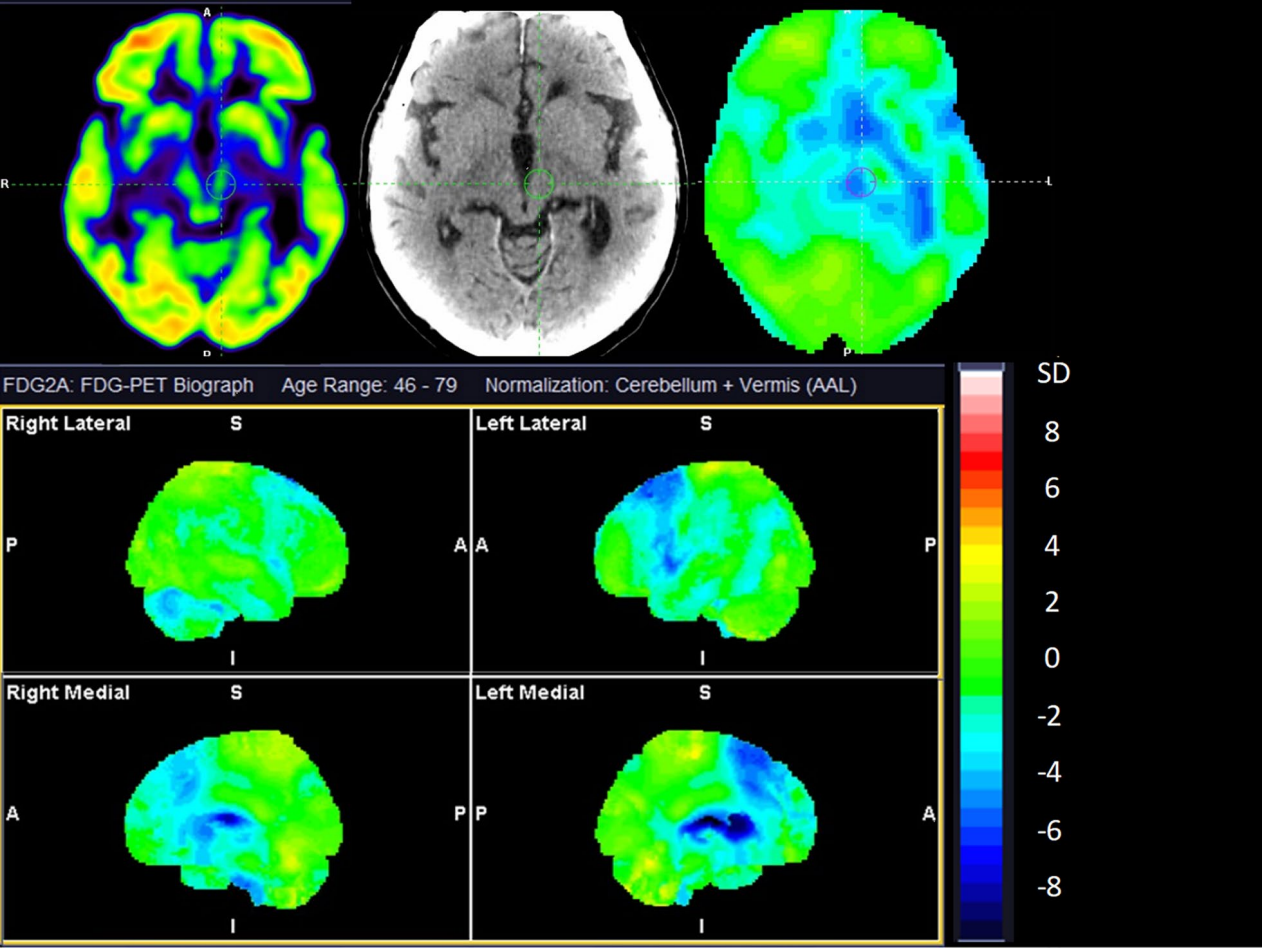
Cerebral [^18^F]FDG PET of a 73-year-old man with overlapping features of CBD and PSP. Top row show axial sections at the level of the basal ganglia and mesencephalon of [^18^F]FDG, CT, and statistical maps. The bottom rows show statistical surface projections with standard deviations from healthy subjects. Please note the asymmetry and involvement of the basal ganglia pointing towards CBD and the involvement of mesencephalon and mesial frontal cortex pointing towards PSP

We adopted the diagnostic criteria for the subtypes of AP, including PSP [13], MSA [14], DLB/PDD [15] and CBD [16]. We decided to pool PSP and CBD as well as DLB and PDD due to the overlap of clinical presentation and similar neuropathology (Fig. 2).

We classified MSA based on features of parkinsonism (striatonigral degeneration, subgroup MSA-P) or cerebellar dysfunction (olivopontocerebellar atrophy, subgroup MSA-C).

### Imaging methods

Subjects fasted 6 h prior to the PET study. The patients were injected with 199 ± 4.9 MBq of [^18^F]FDG intravenously, followed by a rest period of 60 min. Subsequently, patients underwent a 10 min [^18^F]FDG PET/CT scan in a digital Discovery MI PET/CT (GE Healthcare, Milwaukee, USA) in 3-dimensional mode. The ordered subset expectation maximization (OSEM) algorithm was used for image reconstruction and attenuation correction with CT. The [^18^F]FDG PET acquisition procedures complied with the European Association of Nuclear Medicine guidelines [17].

### Imaging analysis

We used Scenium, SyngoVia (Siemens Healthineers, Erlangen, Germany) to analyse the images of the included patients. A nuclear medicine specialist with more than 10 years of experience in PET neuroimaging (LM) classified the scans as MSA-C, MSA-P, CBD, PSP, DLB, or non-AP, based on previously published disease-specific [^18^F]FDG features [18]. Briefly, a reduced [^18^F]FDG uptake in the cerebellum would suggest a potential diagnosis of MSA-C. MSA-P was considered when there was a symmetric or slightly asymmetric reduction in activity uptake in the posterior putamen. This suspicion was strengthened if both the posterior putamen and cerebellum showed decreased activity uptake with an absence of cortical involvement. CBD was considered if an asymmetrical reduction in frontoparietal activity was observed, alongside involvement of the ipsilateral basal ganglia and mesencephalon. For PSP, a more symmetrical reduction in activity uptake in the mesial and lateral frontal cortex, basal ganglia, and particularly the mesencephalon was indicative. DLB was typically characterized by a reduction in parietotemporooccipital activity uptake, along with the presence of a cingulate island sign. The reader was blinded to all clinical information during the classification process except for previous neuroimaging, if available. Thus, MRI scans and results from dopamine transporter imaging were visually interpreted without access to clinical data and used for reading of the [^18^F]FDG PET. In cases of uncertainty regarding the classification of scans, the nuclear medicine specialist would make a decision based on the best professional judgment to prevent any instances of missing data.

### Reference standard

We used the clinical diagnosis as the reference standard, based on the international consensus criteria^13−16^, all clinical information, test results and initial results from the cerebral [^18^F]FDG. A medical student (NJH) reviewed the electronic patient journal and reviewed diagnoses, medications, treatment plans, and progress plans recorded at the Department of Neurology, Copenhagen University Hospital Bispebjerg, from the time of the initial cerebral [^18^F]FDG PET up to June 2021. If the diagnosis was unclear based on the medical record, a neurologist with more than 10 years of experience in movement disorders (AH) reviewed the journal for a final diagnosis. In cases of uncertainty regarding the final diagnosis, the neurologist would make a decision based on the best professional judgment.

### Statistical analysis

We assessed the sensitivity, specificity, and both positive and negative predictive values (PPV and NPV) by comparing the classification using [18F]FDG PET with the reference standard. For robust results, the diseases were divided into four groups: MSA, 4R-tauopathies (CBD/PSP), DLB/PDD, and non-AP. Confidence intervals were calculated using R statistical software (R Core Team, 2017; R Foundation for Statistical Computing, Vienna, Austria; https://www.R-project.org). We followed the standards for reporting diagnostic accuracy studies (STARD).

## Results

After screening patient referrals, we excluded 200 [18F]FDG PET scans due to suspicions other than AP, such as cancer, dementia, or Alzheimer’s disease. Of these, 14 were follow-up cerebral [^18^F]FDG PET or had major MRI findings precluding a reliable AP diagnosis (Fig. 1) leaving a total of 156 patients referred for a cerebral [^18^F]FDG PET for suspicion of AP. Please refer to Table 1 for demographics of included patients. The patients had a median follow-up time of 23 months (range 6–72 months) after the cerebral [^18^F]FDG PET and 24 patients died in the study period. The follow-up diagnosis of the patients were MSA (n = 20, of which MSA-C (n = 5) and MSA-P (n = 15), 4R-tauopathies (n = 68), of which CBD (n = 23) and PSP (n = 45), DLB/PDD (n = 26), and non-AP (n = 42). Unfortunately, the patients did not have a histopathological diagnosis. [^18^F]FDG PET correctly classified a total of 116 (74%) patients into the four groups: MSA: 20, DLB/PDD: 21, 4R-tauopaties: 42, and non-AP: 33. Please refer to Tables 2 and 3 for overview of the classifications and subclassifications relative to follow-up. The sensitivity/specificity for AP was 0.82 (0.73–0.88)/0.79 (0.63–0.88), MSA was 1.00 (0.83–1.00)/0.91 (0.85–0.95), DLB/PDD was 0.81 (0.61–0.93)/0.97 (0.92–0.99), and 4R-tauopathies 0.62 (0.49–0.73)/0.97 (0.90–0.99) (Table 4).

**Table 1:** Subject demographics

Follow up diagnosis	N	M:F	Mean age ± standard deviation	Median follow-up time (from PET to follow-up/ months (range)	Number with previous DAT imaging	Number with previous MRI	Number with previous MoCa test	Mean MoCa score ± standard deviation
MSA	20	5:15	63.4 ± 3.8	21 (4–40)	18	17	17	22.8 ± 4.6
DLB/PDD	26	16:10	71.7 ± 3.5	22 (7–42)	18	20	12	21.9 ± 3.8
CBD	23	12:11	74.5 ± 2.0	24 (10–72)	23	19	13	23.2 ± 3.7
PSP	45	25:20	72.2 ± 2.1	24 (6–50)	41	41	29	21.7 ± 4.7
Non-AP	42	32:10	70.9 ± 2.8	23 (15–37)	30	40	28	21.9 ± 5.7

M, males; F, females; MSA, multiple system atrophy; DLB/PDD, Dementia with Lewy bodies/Parkinson’s disease with dementia; CBS, corticobasal syndrome; PSP, progressive supranuclear palsy; AP, atypical parkinsonism

**Table 2:** Classifications

Follow up diagnosis	Imaging test outcome N = 156				Total
	MSA	DLB/PDD	4R-Tauopaties (CBD/PSP)	Non-AP	
MSA	20	0	0	0	20
DLB/PDD	0	21	0	5	26
4R-Tauopathies (CBD/PSP)	8	2	42	16	68
Non-AP	4	2	3	33	42
Total	32	25	45	54	156

Rows with the follow-up diagnosis and columns with the corresponding imaging classification. MSA, multiple system atrophy; DLB/PDD, Dementia with Lewy bodies/Parkinson’s disease with dementia; CBD, corticobasal degeneration; PSP, progressive supranuclear palsy; AP, atypical parkinsonism

**Table 3:** Subgroup classifications

Follow up diagnosis	Imaging test outcome N = 42		Follow up diagnosis	Imaging test outcome N = 20	
	CBD	PSP		MSA-P	MSA-C
CBD	7	4	MSA-P	12	3
PSP	3	28	MSA-C	0	5

Left: subgroup classification of the 4R-Tauopathies. Right: subgroup classification of MSA. Rows with follow-up diagnosis and columns with the corresponding imaging classification

CBD, corticobasal degeneration; PSP, progressive supranuclear palsy; MSA-P, multiple system atrophy with striatonigral degeneration; MSA-C, multiple system atrophy with cerebellar dysfunction

**Table 4:** Sensitivities and specificities

Follow-up diagnosis	Sensitivity (95% CI)	Specificity (95% CI)	Positive predictive value (95% CI)	Negative predictive value (95% CI)
AP	0.82 (0.73–0.88)	0.79 (0.63–0.88)	0.91 (0.85–0.95)	0.61 (0.51–0.70)
MSA	1.00 (0.83–1.00)	0.91 (0.85–0.95)	0.62 (0.49–0.74)	1.00 (0.97–1.00)
DLB/PDD	0.81 (0.61–0.93)	0.97 (0.92–0.99)	0.84 (0.66–0.93)	0.96 (0.92–0.98)
4R-tauopathies (CBD/PSP)	0.62 (0.49–0.73)	0.97 (0.90–0.99)	0.93 (0.82–0.99)	0.77 (0.71–0.82)
NON-AP	0.79 (0.63–0.88)	0.82 (0.73–0.88)	0.61 (0.51–0.70)	0.91 (0.85–0.95)

AP, atypical parkinsonism; MSA, multiple system atrophy; DLB/PDD, Dementia with Lewy bodies/Parkinson’s disease with dementia; CBD, corticobasal degeneration; PSP, progressive supranuclear palsy

## Discussion

Our study demonstrate that cerebral [^18^F]FDG PET provides a moderate to high diagnostic sensitivity and specificity for differentiation between subtypes of AP with significant sensitivity (> 80%) and specificity (> 90%). However, the sensitivity falls to approximately 62% for 4R-tauopathies, mirroring the known diagnostic challenges of these conditions. These findings support earlier studies that showed the efficacy of [^18^F]FDG PET in differentiating AP subtypes, which reported similar levels of sensitivity and specificity (> 85%) [10, 11].

Challenges arise with CBD due to its overlapping metabolic pattern with PSP, which was identified in a prior prospective study that reported a low PPV (67%) for CBD [12]. This is likely because CBD and PSP share certain neuropathological features, such as basophilic inclusions and distinct cytoskeletal abnormalities, making them difficult to differentiate [19]. Our study’s moderate sensitivity in diagnosing 4R-tauopathies, particularly CBD, aligns with the general difficulties encountered in their accurate clinical diagnosis, as reported in previous studies [20, 21].

The strengths of our study include the inclusion of a diverse patient population consecutively referred for cerebral [^18^F]FDG imaging due to suspected atypical parkinsonism, thereby reflecting real-world clinical practice. We also adhered to the standards for reporting diagnostic accuracy studies.

Limitations of our study include potential bias of the reference standard. The clinical diagnosis might have been influenced by neuroimaging results, challenging the independence of reference standards from the test results. A follow-up consultation would be necessary to assess the neurological status and clinical appearance without access to imaging data, which would significantly reduce the sample size of the most ill subjects, as 24 patients died in the study period, and a significant portion of the patient population, due to the severity of their illness, would be unable to participate. Another limitation involves the lack of post-mortem histopathological diagnosis, which raises questions about the follow-up diagnosis's robustness, especially in uncertain clinical cases. However, histopathological diagnosis is rarely performed, and selective inclusion of patients with a histopathological diagnosis would not be possible with the present material. In a post-mortem sample of 25 patients with MSA symptomatology included at our Neurology department before 2015, 22 showed MSA pathology post-mortem, 2 showed other pathology, and one was inconclusive (unpublished data). Further limitations include the access to previous imaging in the diagnostic reading of the [^18^F]FDG PET, which limits the assessment of the independent information by [^18^F]FDG PET. However, clinical [^18^F]FDG PET reading without the access to MRI or other structural information is not relevant according to practise guidelines [11], and DAT scans are shown to be unable to distinguish between parkinsonian syndromes [14] but is likely to have improved the discrimination between parkinsonian syndromes from healthy ageing. Thus, we chose to include previous imaging to reflect daily clinical practice. Additional readers would have strengthen the results and provided measures of interrater variation. A high specificity of [^18^F]FDG PET is believed to be of diagnostic, prognostic, and survival benefit, as is also seen in other patient groups [22]. There are currently ongoing clinical treatment trials for AP subtypes [23], for which an early diagnosis is essential for correct stratification of the patients, ensuring more reliable and robust outcomes. Despite these limitations, our findings reinforce the utility of cerebral [^18^F]FDG PET. Specific tracers for proteinopathies could potentially enhance sensitivity and specificity, as seen with amyloid imaging in Alzheimer’s disease. Promising tracers such as the tau tracer [^18^F]APN-1607 and the novel [^18^F]PI-2620 are under study and could greatly aid in early diagnosis [24, 25]. Unfortunately, an α-synuclein PET tracer is still missing [26]. The mitochondrial translocator protein (TSPO) binding [^11^C]PBR28 shows potential as a neuroinflammation tracer. Recent studies suggest its use as an imaging biomarker for MSA with high sensitivity and specificity [27]. However, the necessity for genotyping for TSPO single-nucleotide polymorphism to exclude potential false negatives remains a hurdle [28]. Future research to examine the role of tau and neuroinflammation PET tracers in diagnosing early-stage AP is greatly encouraged.

## Conclusion

This study found a moderate to high diagnostic accuracy for [^18^F]FDG PET imaging in the diagnosis of AP in a mixed population resembling clinical routine. The results support the additional use of [^18^F]FDG PET for the clinical diagnosis of AP with high specificity. [^18^F]FDG PET may be beneficial for prognosis and supportive treatment of the patients and useful for future clinical treatment trials ensuring correct stratification of patients.

## Data Availability

Anonymised datasets generated during and/or analysed during the current study are available from the corresponding author on reasonable request.

## Abbreviations

AP: Atypical parkinsonism
CBD: Corticobasal degeneration
CI: Confidence interval
DAT: Dopamine transporters
DLB: Dementia with Lewy bodies
FTD: Frontotemporal dementia
IPD: Idiopatic Parkinson’s disease
MoCA: Montreal Cognitive Assessment
MRI: Magnetic resonance imaging
MSA: Multiple system atrophy
MSA-C: Subgroup of MSA with cerebellar dysfunction
MSA-P: Subgroup of MSA with striatonigral degeneration
PDD: Parkinson’s disease with dementia
PET: Positron emission tomography
PSP: Progressive supranuclear palsy
SPECT: Single-photon emission computed tomography
STARD: Standards for reporting diagnostic accuracy studies
[^18^F]FDG: [^18^F]fluoro-2-deoxy-2-d-glucose
4R: Specific 4-repeat
